# Melanoma risk assessment and management: a qualitative study among Australian GPs

**DOI:** 10.3399/BJGP.2021.0668

**Published:** 2022-09-06

**Authors:** Balakumar Anandasivam, Chun Wah Michael Tam, Kevin McGeechan, Karen Price, Katrina McLean, Marguerite Tracy, John Hall, Andrew Knight, Kylie Vuong

**Affiliations:** Port Macquarie Rural Clinical School, Faculty of Medicine, University of New South Wales, Sydney.; School of Population Health, Faculty of Medicine, University of New South Wales, Sydney; director, Primary and Integrated Care Unit, South Western Sydney Local Health District, Liverpool.; School of Public Health, Faculty of Medicine and Health, University of Sydney, Sydney.; Department of General Practice, Monash University, Melbourne; president, Royal Australian College of General Practitioners, Melbourne.; Faculty of Health Sciences and Medicine, Bond University, Robina.; School of Public Health, Faculty of Medicine and Health, University of Sydney, Sydney.; School of Population Health, Faculty of Medicine, University of New South Wales, Sydney.; School of Population Health, Faculty of Medicine, University of New South Wales, Sydney; staff specialist, Primary and Integrated Care Unit, South Western Sydney Local Health District, Liverpool.; School of Medicine and Dentistry, Griffith University, Gold Coast; adjunct associate professor, School of Population Health, Faculty of Medicine, University of New South Wales, Sydney.

**Keywords:** general practitioners, melanoma, qualitative research, risk assessment, primary health care, Australia

## Abstract

**Background:**

Preventive guidelines for melanoma recommend that patients at high risk of melanoma receive targeted screening; however, this requires careful selection of those at high risk. To the authors’ knowledge, there has been no previous research into how all physicians approach the selection and management of high-risk individuals. Melanoma risk-prediction models are available to assist in the identification of high-risk patients but are not routinely used clinically.

**Aim:**

To examine how GPs assessed and managed melanoma risk, and the opportunities for using melanoma risk-prediction models in primary care.

**Design and setting:**

Semi-structured telephone interviews were conducted with 20 Australian GPs.

**Method:**

GPs who had completed a cross-sectional online questionnaire study on melanoma risk were purposively sampled and recruited. Semi-structured telephone interviews were conducted with Australian GPs between 9 July and 10 September 2019. Interviews were audiorecorded, professionally transcribed, and analysed using grounded theory.

**Results:**

Melanoma risk assessment and its management can be understood as a linear workflow consisting of five clinical process domains with patient selection as the entry point. There was variation between GPs on the identification of melanoma risk factors, melanoma risk estimation, management, and patient education because of intuitive and analytical processes guiding risk assessment, and the influence of patient factors. GPs were largely receptive towards melanoma risk-prediction models, sharing facilitators for and barriers to their potential implementation.

**Conclusion:**

Further primary care interventions sensitive to existing workflow arrangements may be required to standardise melanoma risk-assessment and management processes.

## INTRODUCTION

Melanoma is preventable. However, incidence rates continue to increase among fair-skinned populations despite longstanding public education programmes.^1^^–^3 Population-wide screening for melanoma is not supported by the current evidence.^4^^,^^5^ There is agreement across guidelines that patients who are at high risk of melanoma should be identified and receive targeted screening. Yet, there is poor agreement on what constitutes ‘high risk’.^6^^–^8

In Australia, where melanoma incidence is the highest in the world, GPs play a critical role in reducing melanoma burden by identifying those patients at high risk and in managing the majority of initial diagnoses.^9^^–^12 Clinical guidelines recommend a risk-stratified approach to melanoma prevention.^12^ These guidelines stratify patients into risk levels based on the presence of individual melanoma risk factors. People at average risk receive primary preventive advice, those at increased risk receive primary preventive advice and opportunistic skin checks with a physician, and those at high risk (relative risk >6) receive preventive advice, advice on self-skin-checks, and at least annual skin checks with a physician.^12^

Melanoma risk-prediction models may assist in the accurate identification of high-risk patients by estimating an individual’s overall melanoma risk based on the combination of risk factors present,^13^ with some models showing good discrimination on external validation.^14^^,^^15^ However, current guidelines do not recommend the routine use of melanoma risk-prediction models because of a lack of validated models and prospective evaluation.^12^^,^^16^^,^^17^

Further research to support the identification and targeted screening of high-risk individuals in routine clinical practice is needed.^7^^,^^8^^,^^17^^,^^18^ To the authors’ knowledge, there has been no previous research into how all physicians approach melanoma risk assessment and its management. The aim of the current study was to examine how GPs assessed and managed melanoma risk, and the opportunities for using melanoma risk-prediction models in primary care.

**Table:** How this fits in

Preventive guidelines for melanoma recommend that patients at high risk of melanoma receive targeted screening; however, this requires careful selection of those at high risk. This study indicates that there is variation in how GPs assess and manage melanoma risk across five clinical process domains, with greatest variation among GP participants on how they estimated overall melanoma risk. Further interventions may be required to standardise these processes such as the implementation of risk-prediction models. If melanoma risk-prediction models are to be used within primary care, it will need to be sensitive to the host setting and the clinical workflow within it.

## METHOD

### Design, setting, and participants

This was a qualitative, descriptive– interpretive study following a grounded-theory approach suggested by Corbin and Strauss to analyse data collected from semi-structured interviews.^19^ This approach is useful for understanding the behaviours, thoughts, and emotions of people within their sociocultural context, in this case, GPs in their clinical practice.^19^ In the current study, the analysis assumed the existence of an explanatory theory that was ‘grounded’ in the data and could be co-constructed through the researcher’s interaction with the data.^20^ It is reported in accordance with the consolidated criteria for reporting qualitative studies.^21^

This research was undertaken in Australia in 2019. The lifetime risk of being diagnosed with melanoma in Australia is estimated to be one in 15 people, with a lifetime melanoma mortality risk of one in 140, making melanoma the third most diagnosed cancer (excluding keratinocyte cancers) and the eighth leading cause of cancer-related death.^22^ Most residents (83%) consulted with a GP at least once during the 2019 Australian financial year.^23^ GPs hold specialist registration in Australia and have important responsibilities in melanoma prevention, diagnosis, management, and follow-up.^24^

Seven of the researchers, including the senior author, are clinical academic GPs, one is a biostatistician with research experience in cancer prevention in primary care, and the first author is a medical student researcher. The senior author has research experience in cancer prevention and melanoma epidemiology.

Individuals were invited to take part from the 136 Australian GPs who had completed a cross-sectional questionnaire-based study on the topic of melanoma risk assessment. The cross-sectional study participants were recruited between June and August 2019 from GPs Down Under, a Facebook group comprising over 6000 authenticated Australian and New Zealand GPs.^25^ In this cross-sectional study, the extent of agreement between unassisted clinician self-reported- and model-generated melanoma risk predictions was assessed. GPs who had previously received a diagnosis of melanoma were ineligible because they were at high risk of melanoma and were excluded from the cross-sectional study.

A total of 44 GPs expressed interest in participating in this qualitative study by providing their contact details. From this pool, potential participants were purposively sampled for diversity in geographic location of practice, training pathways, registration type, and clinical experience in melanoma. Invitations were sent by email in sequential recruitment rounds until theoretical saturation was achieved in analysis. Non-responders were sent a reminder email after 2 weeks. Reasons for non-participation were not sought. Verbal consent was obtained from all participants before the interviews.

### Data collection

Semi-structured individual telephone interviews were conducted from 9 July to 10 September 2019. The semi-structured interviews collected open-ended data through dialogue guided by a flexible interview guide with follow-up questions, probes, and comments.^26^ The interview guide (see Supplementary Box S1) was developed through discussion and trial interviews with a convenience sample of four GPs.

The participants had no established relationship to the interviewer before study commencement. Interviews were conducted by one investigator (the first author), a medical student researcher, trained and supervised by the second author, a GP with qualitative research expertise. Interviews were digitally audiorecorded, transcribed verbatim by a professional service, checked for errors, and de-identified before being uploaded into QSR NVivo (version 12) software for analyses. Participants were not invited to review their interview transcripts. Hand-written and typed field notes were taken during and immediately after each interview. Participant sociodemographic and professional details were obtained from the questionnaire items completed by the participants as part of the cross-sectional study.

Theoretical saturation was assessed for at weekly team meetings by considering the fit of new data into the existing analyses.^27^^,^^28^ It was likely that the analysis was approaching saturation by the 15th interview; however, several further interviews were undertaken to increase participant variation and to specifically explore aspects of the developing model. It was agreed that theoretical saturation had occurred after the 20th interview, and recruitment was halted.

### Data analysis and interpretation

The first and second author performed in vivo open coding on the earlier interview transcripts to produce the verbatim codes. Four investigators assisted with the axial coding process whereby the verbatim codes were inductively abstracted and organised into concepts, categories, and themes at weekly meetings. Emerging codes were tested using constant comparative analysis, further explored, and tested through the theoretical sampling of later interviewees, and selective coding of their interview transcripts. During team meetings, concept mapping and reflexivity through identifying, discussing, and challenging established assumptions was essential to developing the final theoretical model. QSR NVivo (version 12) software was used to facilitate the analysis.

## RESULTS

Among the 29 GPs approached during sequential recruitment rounds prior to achieving data saturation, 20 agreed to participate in telephone interviews. The age of participants ranged from 26 to 66 years, with an even split between males and females. Clinical experience as a GP ranged from 1 to 37 years. Participants were located across five (of a total of eight) states/territories within Australia, with all but two living in a major city. The interviews took on average 28 min to complete, ranging from 18 to 34 min. Table 1 provides demographic and training characteristics of each participant in more detail.

**Table 1.** Participant characteristics

**Participant number**	**Age, years**	**Sex**	**State**	**Principal location of work**	**Registration status**	**Years worked in general practice**	**Number of half-day clinical work per week**	**Work in skin cancer clinic**	**Country where primary medical qualification was attained**	**Attainment of post-graduate skin qualification**
1	32	M	VIC	Metropolitan	Specialist GP	5	10	Yes	Australia	Yes, Master’s level
2	38	M	VIC	Metropolitan	Specialist GP	3	6	No	Australia	None
3	57	M	QLD	Metropolitan	Specialist GP	32	6	No	Australia	None
4	54	M	QLD	Regional town	Specialist GP	25	9	No	Australia	None
5	57	F	NSW	Metropolitan	Specialist GP	30	6	No	Australia	None
6	34	F	WA	Metropolitan	Specialist GP	5	4	Yes	Australia	None
7	33	F	VIC	Metropolitan	Specialist GP	5	8	No	Australia	None
8	33	M	WA	Metropolitan	Specialist GP	5	2	No	Australia	None
9	61	F	VIC	Metropolitan	Specialist GP	35	9	No	Australia	None
10	52	F	WA	Remote community	Specialist GP	26	4	No	Australia	None
11	56	M	VIC	Metropolitan	Specialist GP	29	10	No	Australia	None
12	41	M	NSW	Metropolitan	Specialist GP	10	9	No	Australia	None
13	40	F	NSW	Metropolitan	Specialist GP	14	5	No	Australia	None
14	30	F	NSW	Metropolitan	Specialist GP	5	5	No	Australia	Yes, certificate level
15	51	F	VIC	Metropolitan	Specialist GP	19	8	No	Australia	Yes, Master’s level
16	35	F	QLD	Metropolitan	Specialist GP	2	7	No	New Zealand	Yes, certificate level
17	66	M	NSW	Metropolitan	Specialist GP	37	8	No	Australia	Yes, certificate level
18	35	M	ACT	Metropolitan	Trainee	3	9	No	Australia	None
19	56	M	QLD	Metropolitan	Specialist GP	25	8	No	UK	None
20	26	F	NSW	Metropolitan	Trainee	1	10	No	Australia	None

*ACT = Australian Capital Territory. NSW = New South Wales. QLD = Queensland. VIC = Victoria. WA = Western Australia.*

**Figure 1.**
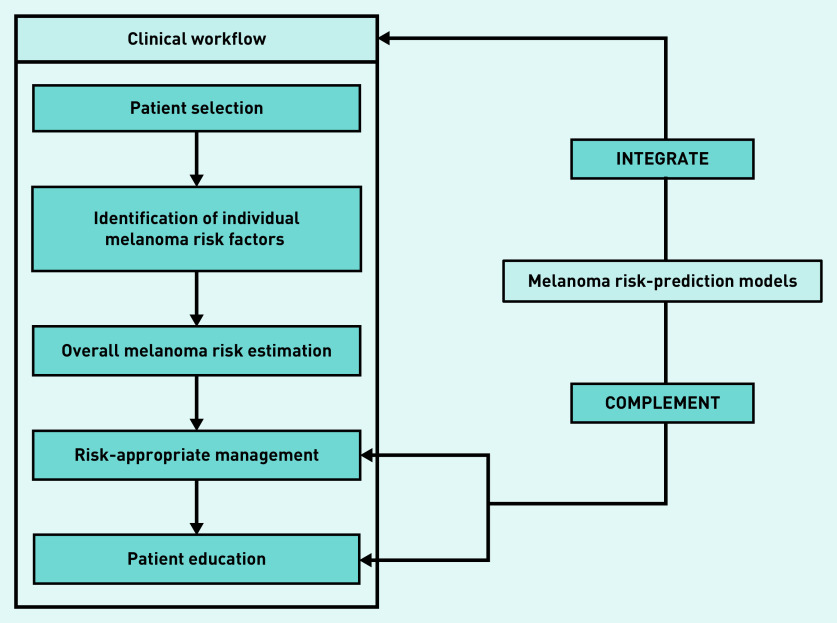
*Explanatory model of how GPs conceptualise melanoma risk assessment and management, and opportunities for using melanoma risk-prediction models.*

The explanatory model that emerged demonstrated that GP assessment of melanoma risk and its management can be understood as a linear workflow consisting of five clinical process domains starting with patient selection as the entry point based on the clinical context (Figure 1). The GPs largely welcomed the role of melanoma risk-prediction models within clinical practice, sharing facilitators and barriers to them integrating into the existing clinical workflow and complementing risk-appropriate management and patient education.

### Patient selection

The participants framed melanoma risk assessment as initiated by two main clinical contexts. One involves the opportunistic assessment of melanoma risk as part of a general preventive health assessment:

*‘… it’s generally part of my whole preventative care screen … highlighting that they need a skin check in other consults and then making them book appointments specifically to come back to me for the skin check.’*

(Participant 20, 1 year’s experience in general practice)

The other involves the assessment of melanoma risk following specific skin cancer-related prompts such as a skin check appointment, a suspicious skin lesion, or a discussion of skin cancer risk factors:

*‘I do a number of formal skin checks, people walk in to me specifically to have a skin check, so that’s a formal part of that consult, but if somebody just comes in just concerned about one mole, then I will go through that list anyway, for that one mole.’*

(Participant 15, 19 years’ experience in general practice)

*‘… if somebody said to me “Oh, you know, I’ve got a lesion” but before I look at it I would be thinking, well, what factors would make me more suspicious or less suspicious before looking at the lesion.’*

(Participant 8, 5 years’ experience in general practice)

### Identification of individual melanoma risk factors

Most participants described verbally running through an informal checklist of risk factors and protective factors with each patient as part of their clinical assessment.

Risk factors and protective factors mentioned by the participants on prompting included: patient age and sex; phenotypic features including eye and hair colour, naevi density, and the presence of atypical naevi and actinic damage; past ultraviolet exposure including the country most lived in, actinic damage, occupational and recreational exposure, and sunburn history; personal skin cancer history including the number of skin excisions, the number of keratinocyte cancers, and melanoma; a family history of melanoma; and immunosuppression; as well as sun-safe practices:

*‘… it’s Fitzpatrick skin types, personal and family history of skin cancers, or other kinds of cancers. Whether they have had outdoor jobs or hobbies. If they got regular sunburns in childhood … if they have used sun protection or sunscreen … ’*

(Participant 20, 1 year’s experience in general practice)

### Overall melanoma risk estimation

The participants considered the patient’s set of risk factors and protective factors to stratify them to a risk level. This relied on both intuitive and analytical processes that were supported by the participant’s knowledge of clinical guidelines, skin cancer training, and clinical experience. No participants reported using a melanoma risk-prediction model in their routine clinical practice:

*‘The skin cancer college courses they’ve highlighted the risk factors that we need to highlight. So I’ve basically based my practice on that … I don’t actually follow a pathway as such. A lot of it is general judgement and assessments.’*

(Participant 16, 2 years’ experience in general practice)

*‘I often turn to the Cancer Council’s guide on melanoma and other skin cancer, that is probably my most useful resource in terms of making decisions and making assessments.’*

(Participant 8, 5 years’ experience in general practice)

On further probing, three analytical processes were used to varying degrees. First, many participants described the importance of recognising the presence of major risk factors, such as family and sun-exposure history, which immediately qualifies the patient into a higher risk group:

*‘I guess it depends on which risk factor. If they’ve got a family history of melanoma then I would put them straight into the high* [risk level] *.’*

(Participant 14, 5 years’ experience in general practice)

Second, some participants described the use of the total number of risk factors identified as a proportional measure of risk:

*‘I have a proforma history that I’ve been doing for so long. I just go through their history when they come in for their skin check … with each one that they answer in the positive to, then my concern about their risk of melanoma goes up.’*

(Participant 15, 19 years’ experience in general practice)

Third, a few participants described the moderation of the effect of certain risk factors in the setting of protective factors in the same patient:

*‘A Fitzpatrick one or two skin probably doesn’t impress me … if they’ve never developed a problem.’*

(Participant 4, 25 years’ experience in general practice)

Participants then described allocating patients into a diverse number of risk levels. Some participants divided their patients into binary risk levels:

*‘The risks stratification’s pretty crude. There’s the basket called low risk and then there is everything else. Unless you are answering no to all those questions that I gave before, then you are not low risk.’*

(Participant 18, 3 years’ experience in general practice)

Other participants stratified the patients into three or four risk levels. The number of risk levels conceptualised by a participant seemed closely related to the number of conceptualised management pathways:

*‘I’m just trying to work out whether they’re at low, medium, or high risk as a background thing, and that helps me also advise them on how often they should be having a skin check and how important it is for them to be taking outside sun precautions.’*

(Participant 14, 5 years’ experience in general practice)

### Risk-appropriate management

The management options included sun-safe education, skin surveillance, specialist referral, and lesion excision. The chosen management was commensurate on the patient’s overall melanoma risk level as estimated by the GP, patient factors, as well as physician factors such as confidence, expertise, and access to certain technologies and skin cancer services:

*‘If I think they’re at high risk, they may even need six-monthly or yearly skin checks, otherwise, every one to two years I think is reasonable.’*

(Participant 12, 10 years’ experience in general practice)

*‘I think it just helps me because often you do see a lesion and you’re thinking, “Oh, it looks benign but I’m not one hundred per cent sure”, and I think having a background risk helps you in decision making as to whether you refer it, or whether you biopsy it yourself, or whether you’re happy to observe it.’*

(Participant 14, 5 years’ experience in general practice)

*‘I think it’s probably the number of risk factors … I guess it also depends on how likely they are* [the patients] *to be able to come in if there’s something wrong.’*

(Participant 13, 14 years’ experience in general practice)

### Patient education

The GP participants described communicating melanoma risk to patients in terms of the individual melanoma risk factors identified or the overall melanoma risk level estimated:

*‘I would probably say to them look, you’ve got this risk factor and that risk factor, and we should be checking you more frequently to make sure that we can pick early change and get things before they become major issues.’*

(Participant 5, 30 years’ experience in general practice)

*‘I’d say broadly like increased risk, and normally emphasis that it is preventable … I don’t have patients asking for that type of specific information, wanting a percentage figure, most of them are fairly satisfied.’*

(Participant 3, 32 years’ experience in general practice)

The communication of risk was reported to be individualised based on the patient’s identified risk factors, health literacy, and perceived concern. It was sometimes numerically supported with the relative risks conferred by individual risk factors or by comparing the absolute lifetime risk of developing melanoma in the general population to other events:

*‘I’ve got some stuff in my work space that talks about each of those things and how much additional risk they might confer … I’ll use that not just in words, but I’ll actually go through those risks with them.’*

(Participant 6, 5 years’ experience in general practice)

*‘You can try* [to] *compare it to other risks that they might be facing. You try to balance it; you don’t want to have them in absolute fear, but you also want them to responsibly manage the risk.’*

(Participant 3, 32 years’ experience in general practice)

### Opportunities for using melanoma risk-prediction models

Most participants were receptive towards potentially using melanoma risk-prediction models in melanoma risk assessment and management. They felt it could:

- integrate into the clinical workflow; and
- complement downstream clinical process themes including risk-appropriate management and patient education (Figure 1).

#### Integration into existing clinical workflow

Participants described the potential benefit of having a melanoma risk-prediction model as part of the electronic health record for easy access:

*‘When I do find* [risk-prediction models] *useful they are easily accessible for me, because I have things like the cardiovascular risk and the CHA2 DS2 -VASc score calculator on my computer anyway, so I’ve got those calculators at my fingertip.’*

(Participant 19, 25 years’ experience in general practice)

Intelligent data automation was suggested to bypass user interface inefficiencies:

*‘If it were to auto populate … automatically calculate the score for you and maybe something about them then yes, absolutely we’ll use it.’*

(Participant 16, 2 years’ experience in general practice)

The participants also projected other settings in which a self-assessable melanoma risk-prediction model could be used, such as in the waiting room (on a tablet computing device) or outside the practice, as potentially beneficial to both the patient and physician:

*‘… it’s beneficial for patients to have the opportunity to assess their own risk.’*

(Participant 3, 32 years’ experience in general practice)

*‘If patients use it as a self-assessment tool, then it’s not going to be a cognitive burden on the doctor.’*

(Participant 18, 3 years’ experience in general practice)

If a melanoma risk-prediction model were to be used outside the practice, these participants considered it important for it to complement and not replace medical advice as there is a risk that patients could misinterpret the results:

*‘… you’d want to be making sure that it’s in the context of them about to walk into the doctor’s or there’s an opportunity for them to discuss that risk afterwards. I guess it has the danger of falsely reassuring that’d be my potential worry.’*

(Participant 6, 5 years’ experience in general practice)

#### Complement downstream clinical process themes

The participants shared opportunities for a melanoma risk-prediction model to complement patient education, suggesting absolute risk levels should be delivered in easily understood formats, such as in colourised tables and graphs. However, most participants preferred relative risk numbers when educating patients:

*‘I think absolute* [risk] *is more helpful for a clinician when they’re thinking about risk, but I think relative risk is more useful when you’re trying to influence behaviour change.’*

(Participant 1, 5 years’ experience in general practice)

Participants indicated that risk estimates should be paired with evidence-based management guidelines to support both risk-appropriate management, particularly for less experienced physicians, and patient education:

*‘As long as it went if you have this, this is the action you take, that could be helpful. I think that would be particularly helpful for GP registrars starting out.’*

(Participant 13, 14 years’ experience in general practice)

*‘I think having that really concrete information really helps patients be better with the preventative health. I think the vaguer we are, the less likely they are to adhere.’*

(Participant 7, 5 years’ experience in general practice)

However, a few participants expressed that such models may not substantially change or improve current practices regarding management recommendations:

*‘I think the fact that there are relatively few courses of possible action when you identify someone at higher risk means that precisely estimating the risk probably doesn’t feed into changes in clinical practice.’*

(Participant 4, 25 years’ experience in general practice)

Some participants expressed possible shortcomings of a melanoma risk-prediction model in regard to its impact on patient-centred management, and its utility in motivating behavioural changes in patients:

*‘… at the end of the day you’re treating a patient; you’re not treating a risk assessment on the screen and the risk assessment’s a good tool, it can tell you if it’s low or high risk, but you shouldn’t just go by that. It should also be guided by what you think the patient in front of you will actually do.’*

(Participant 20, 1 year’s experience in general practice)

*‘If there’s a modifiable risk factor* [in the model] *, and they can see a comparison, sometimes that can be a motivating factor for them to change. But I only see the utility in it being a motivating factor for them to change.’*

(Participant 13, 14 years’ experience in general practice)

## DISCUSSION

### Summary

To the authors’ knowledge, this is the first in-depth study to examine how all physicians assessed and managed overall melanoma risk. Five clinical process domains were identified, with patient selection for melanoma risk assessment as the entry point. There was variation between physicians on the identification of melanoma risk factors, melanoma risk estimation, management, and patient education because of intuitive and analytical processes guiding risk assessment, and the influence of patient factors. GPs were largely receptive towards the role of melanoma risk-prediction models, sharing facilitators and barriers to them integrating into current clinical practice, specifically, in terms of them improving existing clinical workflow, and complementing risk-appropriate management and patient education.

### Strengths and limitations

The strengths of this study include the recruitment of volunteer GPs from across Australia who responded to a questionnaire on melanoma risk; it is likely that these participants have a greater interest in assessing and managing melanoma risk in their patients, including the various melanoma risk-assessment methods. A grounded-theory approach was used, which allowed for the robust development of a possible explanatory model. The study had sufficient data to reach theoretical saturation.

The findings of the current study should be interpreted in the context of several potential limitations. Although the study sampled for variation, GPs-in-training, GPs working in rural and remote areas, and overseas trained GPs were not well represented. Finally, in some of the interviews the participants were told that members of the research team had developed a melanoma risk-prediction model. It is plausible that social desirability bias may have led to more favourable views on the possible use of melanoma risk-prediction models; however, those participants who were not told also shared favourable views.

### Comparison with existing literature

Reforms in primary care, in common with other health settings, have largely focused on disease management over prevention and there is a lack of consistency in risk-assessment practices.^29^ Although Australian GPs manage melanoma frequently, there are no structured procedures to initiate patients for melanoma risk assessment. The GP participants in this study demonstrated high levels of melanoma risk-factor knowledge identifying important melanoma risk factors to guide the identification of patients at high risk and used these factors to stratify management based on risk, which is congruent with preventive guidelines.^7^^,^^12^ Many participants also reported challenges in defining the threshold for high melanoma risk in both absolute and relative terms. This was similar to the phenomenon reported among Canadian primary care physicians on definitions for high cardiovascular disease risk.^30^

There was greatest variation among GP participants on how they estimated overall melanoma risk. Some of the participants in the current study reported that they primarily used intuitive processes, whereas others used analytical processes to identify major melanoma risk factors or the total number of melanoma risk factors with moderation by protective factors.^31^^–^33 This is not surprising as the preventive guidelines provide limited information on how to combine individual melanoma risk factors to estimate overall risk.^7^ In 2015, a prospective Canadian study using real patients found that 29% of physicians used subjective clinical judgement to assess disease risk compared with 12% who counted the number of risk factors to assess disease risk.^34^

The participants in this study described risk stratification occurring with two, three, or four melanoma risk levels compared with most international guidelines describing binary risk levels and the Australian preventive guidelines for GPs describing three risk levels.^7^^,^^12^

Australian GPs are familiar with using risk-assessment models in the clinical setting and delivering risk-based management.^35^ Previously, the authors of the current study have shown that real-time model-generated melanoma risk predictions and tailored prevention advice are associated with better sun-protection behaviours in the intervention patients compared with usual care in Australian general practices.^36^ In UK studies, model-generated melanoma risk predictions have been feasible and acceptable among patients attending general practices.^37^^,^^38^ However, there are no melanoma risk-prediction models in routine clinical use in Australia or internationally.

The GP participants in the current study, similar to physician participants in cardiovascular disease and other cancer studies, had preferences for a melanoma risk-prediction model that can be integrated into electronic record systems,^39^^–^42 which is self-assessable,^43^ presents risk estimates in both numerical and visual forms,^39^^,^^42^^,^^44^ pairs risk estimates with evidence-based management guidelines,^39^^–^41 and incorporates patient factors and motivates behavioural changes in patients.^45^^,^^46^ The GP participants in the current study also expressed potential barriers to the routine use of risk-prediction models in primary care, which are similar to previous findings regarding decision support aids for physicians. They expressed several possible shortcomings of risk-prediction models on workflow including accessibility issues and documentation time,^47^^–^49 being less useful for more experienced doctors,^50^ and not significantly changing management recommendations.^47^

### Implications for practice

The current study indicates that there is variation in how GPs assess and manage melanoma risk across five clinical process domains, with greatest variation among GP participants on how they estimated overall melanoma risk. Further interventions may be required to standardise these processes. If melanoma risk-prediction models are to be successfully implemented within primary care, they will need to be sensitive to the host setting and the clinical workflow within it.
